# Malposition of Cage in Minimally Invasive Oblique Lumbar Interbody Fusion

**DOI:** 10.1155/2018/9142074

**Published:** 2018-07-11

**Authors:** Chaiwat Kraiwattanapong, Vanlapa Arnuntasupakul, Rungthiwa Kantawan, Gun Keorochana, Thamrong Lertudomphonwanit, Pakkanut Sirijaturaporn, Methawut Thonginta

**Affiliations:** ^1^Department of Orthopaedics, Ramathibodi Hospital, Mahidol University, Bangkok, Thailand; ^2^Department of Anesthesiology, Ramathibodi Hospital, Mahidol University, Bangkok, Thailand; ^3^Department of Nursing, Ramathibodi Hospital, Mahidol University, Bangkok, Thailand

## Abstract

**Introduction:**

Minimally invasive oblique lumbar interbody fusion is one of the novel lateral lumbar interbody fusion techniques for which the successful early results have been reported. However, new complications were increasingly reported from ongoing studies.

**Case Presentation:**

We report a case of an unusual complication of minimally invasive oblique lumbar interbody fusion associated with contralateral nerve root compression due to deep and posterior position of polyetheretherketone cage and discussion of the operating technique for repositioning polyetheretherketone cage.

**Conclusion:**

Malposition of polyetheretherketone cage can cause contralateral nerve root compression and neurological complication. The surgical technique to proper pull the polyetheretherketone cage back into the acceptable position should be considered and well prepared.

## 1. Background

Minimally invasive lateral lumbar interbody fusion has gained popularity because of several advantages such as less blood loss, less tissue dissection, larger footprint of implant, maximizing load bearing on the cortical bone, and increasing more lordosis of the lumbar spine [1–4].

Minimally invasive oblique lumbar interbody fusion (MIS-OLIF) is one of the novel lateral lumbar interbody fusion techniques. This technique allows access to the intervertebral disc of lumbar spine via retroperitoneal space, between great vessels and psoas muscle. Mayer first described this minimally invasive anterior to psoas muscle technique for lumbar interbody fusion in 1977 [5]. Davis et al. reported an anatomical study of the oblique corridor at each lumbar disc level between the psoas muscle and great vessels and found the potential of the MIS oblique retroperitoneal approach to the L2–S1 discs [6]. Molinares et al. studied 133 MR images of the lumbar spine and reported the oblique corridors of L2–S1 discs between the psoas and the aorta or the left common iliac artery in 90% of the studied samples [7]. With the similar approach, but different instruments and implants, several studies of the MIS-OLIF in term of outcomes and complications have been reported. From the previous studies, complication of mini-open and MIS-OLIF range from 3.9%–48.3% [8–16]. Most of them are transient and completely recover by time. Few case reports published the special complications of MIS-OLIF such as ureteral injury, ventral dural injury [17–19]. This article presents an unusual complication of MIS-OLIF associated with contralateral nerve root compression due to deep and posterior position of MIS-OLIF polyetheretherketone (PEEK) cage and discusses the operating technique for removing MIS-OLIF PEEK cage.

## 2. Case Presentation

A 60-year-old woman presented with 1-year history of low back pain with lateral aspect of left leg pain and severe neurogenic claudication. There was no neurological deficit. Plain films showed narrowing of L4-L5 disc space and degenerative spondylolisthesis of L4-L5. MRI of L4-L5 showed a degenerative change of intervertebral disc, severe bilateral foraminal stenosis, and moderate central stenosis.

**Figure 1:**
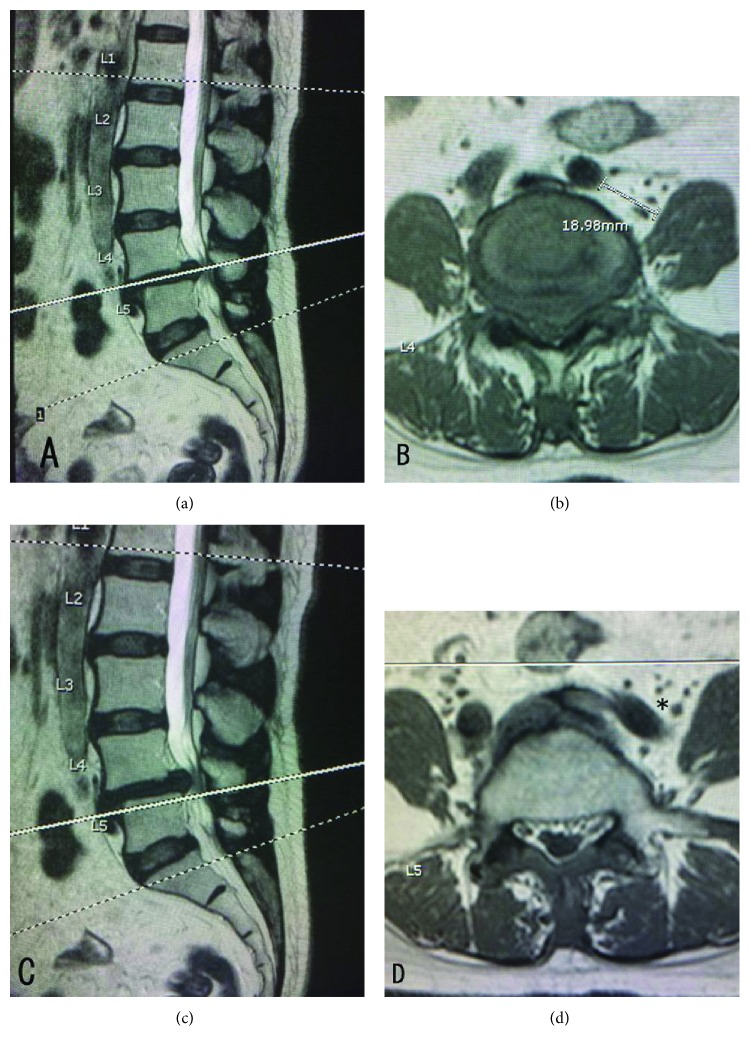
Preoperative MRI of lumbosacral spine T1W image showed prepsoas corridor at the level of intervertebral disc space L4-L5 (a and b) and at the level of upper vertebral body of L5 (c and d). Rising of psoas muscle was shown in (d). Left common iliac artery almost obliterates prepsoas space at level of upper vertebral body of L5 (black asterisk).

On the axial T1W image, the space between the left common iliac artery and the left psoas muscle was 18.98 mm at level of intervertebral disc space L4-L5 which almost obliterated prepsoas space at level of upper vertebral body of L5 (Figure 1). Her symptoms did not improve after conservative treatments. She was scheduled to perform MIS-OLIF with decompressive laminectomy and fixation with cortical bone trajectory screws at L4-L5.

Intraoperatively, after general anesthesia, the patient was put in right lateral decubitus position. Fluoroscopy was used to confirm true AP and true lateral of L4-L5 intervertebral disc space. Lateral retroperitoneal approach to lumbar spine was performed. Guide wire and sequential dilator were placed and then retractor blades and L4 stability pin were placed as usual. Unfortunately, when the retractor blades were distracted, the left common iliac artery was found in the operating field. This could be explained because the left common iliac artery was close to the edge of left psoas muscle as Figure 1.

The retractor blades and stability pin were then removed. The psoas muscle was retracted and guide wire was replaced more posteriorly. The operation was performed as usual and MIS-OLIF PEEK cage (a 6° lordotic-angled CLYDESDALE®) 10 mm × 50 mm was inserted into the intervertebral disc space under fluoroscopic assistance. The final position from fluoroscopy revealed the tantalum marker of MIS-OLIF PEEK cage was pushed more to the right side of the vertebral body. Reposition of MIS-OLIF PEEK cage was not performed at that time. Posterior decompressive laminectomy at L4-5 and cortical bone trajectory screw fixation was then performed in the prone position.

**Figure 2:**
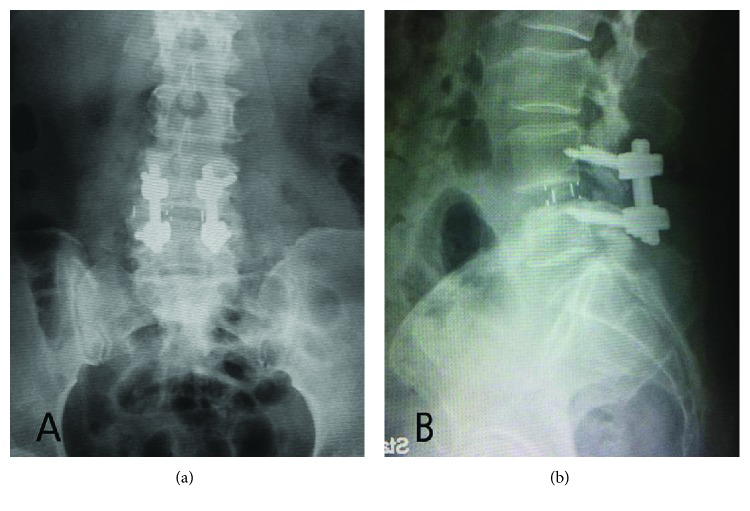
Postoperative plain films of lumbar spine AP and lateral (a and b) showed the MIS-OLIF PEEK cage was placed too deep over edge of right lateral of vertebral body.

Postoperatively, the preoperative pain on the left leg disappeared. However, she developed pain and numbness on her right leg corresponding to L4 dermatome. Plain films showed the position of MIS-OLIF PEEK cage was placed too deep over the edge of the right lateral vertebral body (Figure 2). She then was brought to the operating room to reposition the MIS-OLIF PEEK cage. CT and MRI were not performed before the second operation due to remarkable malposition of the MIS-OLIF PEEK cage with acquired pain and numbness of her right leg.

Intraoperatively, after general anesthesia, the patient was put in right lateral decubitus position. The MIS-OLIF PEEK cage was reached from left lateral approach. The removal tool and slap hammer were attached to MIS-OLIF PEEK cage. The slap hammer was impacted to remove MIS-OLIF PEEK cage. However, the MIS-OLIF PEEK cage could not be repositioned and was attached with vertebral bodies. The cause of this malposition might have been from the compression of posterior cortical bone trajectory screws fixation. The posterior approach was then performed to remove rods from the cortical bone trajectory screws. The removal tool and slap hammer were then attached to the MIS-OLIF PEEK cage. Unfortunately, the MIS-OLIF PEEK cage became stuck and was unmovable.

**Figure 3:**
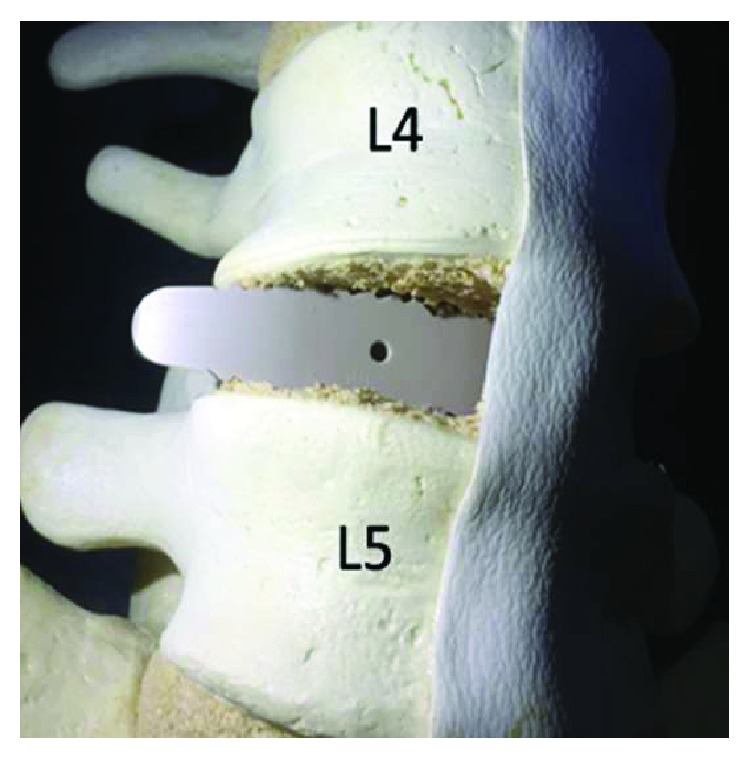
Model picture showed the possibility that MIS-OLIF PEEK cage locked with the vertebral endplates.

**Figure 4:**
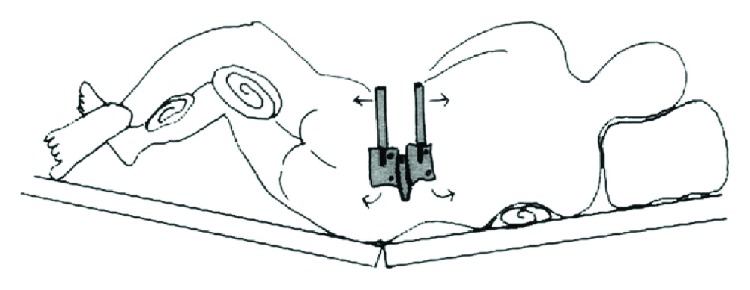
Drawing picture showed the reverse jack-knife position of the patient with distraction of retractor blade pins for loosening the MIS-OLIF PEEK cage form the vertebral endplates.

**Figure 5:**
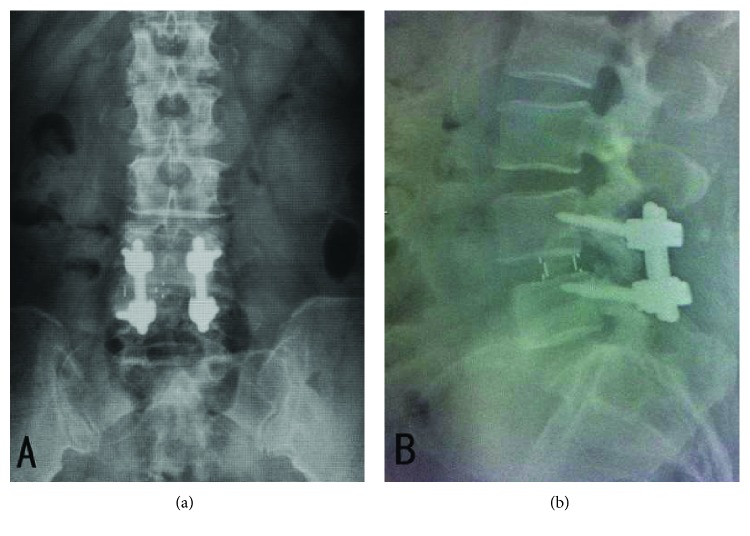
Three months postoperative plain films of lumbar spine AP and lateral (a and b) showed the acceptable position of MIS-OLIF PEEK cage.

The MIS-OLIF PEEK cage teeth might have locked with the right lateral end plates of vertebral bodies (Figure 3). The patient was then placed in reverse jack-knife position for opening of the right lateral intervertebral disc space. Retractor blade pins at L4 and L5 vertebral bodies were gradually distracted (Figure 4). The MIS-OLIF PEEK cage then was gently pulled back and adjusted to a more anterior trajectory to achieve an acceptable position (Figure 5).

Postoperatively, the pain on her right leg disappeared and the numbness was improved. She was able to walk without pain. At 3 months follow-up, her back and leg pain had significantly improved and her right leg numbness disappeared. The Oswestry Disability Index was 64.4 at preoperative time and was 26 and 20 at 2 weeks and 3 months postoperative, respectively.

## 3. Discussion

Minimally invasive lateral interbody fusion is an alternative procedure to the traditional approach for the treatment of degenerative disease of the lumbar spine. Outcome and fusion rate are comparable to traditional interbody fusion with short operating times, minimal blood loss, and few complications [20]. MIS-OLIF is an approach which reaches intervertebral disc through the retroperitoneal space between great vessels and psoas muscle. When we compare transpsoas approach, MIS-OLIF has several advantages such as less invasion of the psoas muscle and lumbar plexus, direct visualization of sensory nerves and important structures. Fujibayashi et al. concluded that MIS-OLIF can be safely performed without using neuromonitoring [21]. However, this novel approach needs more well-designed prospective studies with long-term follow-up. More outcomes and complications of ongoing studies will be published in near future. In this article, we reported the complication which was caused by inappropriate patient selection and inappropriate surgical technique for MIS-OLIF.

This patient might not be a good candidate for MIS-OLIF, because her prepsoas corridor at upper vertebral body of L5 was almost obliterated. Psoas muscle had to be strongly retracted and guide wire had to be placed more posteriorly which resulted in posterior position of MIS-OLIF peek cage. Additionally, her psoas muscle was rising away ventrally from the vertebral body which obstructed the pathway of the entry point to intervertebral disc (Figure 1(d)). Voyadzis et al. reported 3 cases of rising away psoas muscle from the vertebral body which could lead to aborted transpsoas lateral interbody fusion due to pervasive EMG responses throughout the disc space [22]. Currently, there is no report of such complication in MIS-OLIF procedure, but this can cause more difficulty in this situation.

According to the manufacturer, the proper size of the MIS-OLIF PEEK cage should span the entire ring apophysis in order to reach fully across the vertebral body end plate. If the position of MIS-OLIF PEEK cage is more posterior, this step is critical and overhang can create contralateral nerve root compression. Silvestre et al. reported outcomes and complications of OLIF with banana shape TLIF PEEK cage in 179 patients. They reported one case of right L4-5 paresthesia and weakness causing by a prominent of 36 mm long TLIF PEEK cage which compressed the dural sac contralaterally. Due to TLIF PEEK cage which is much smaller than MIS-OLIF PEEK cage, she successfully received revision with placement of shorter TLIF PEEK cage of 30 mm length, but unfortunately she did not recover from her neurological injury [11]. In our case, we used a longer MIS-OLIF PEEK cage (50 mm in length) because this MIS-OLIF PEEK cage was placed more parallel to the posterior cortex of the vertebral body. Papanastassiou et al. reported two cases of contralateral femoral nerve compression after extreme lateral interbody fusion (XLIF). One of them was caused by a displaced endplate fracture fragment and another was caused by a far-lateral herniation. Nerve root decompressions were performed, and patients then experienced resolution of their symptoms [23]. In our case, the deep and posterior MIS-OLIF PEEK cage position caused the compression of the nerve plexus, so we then tried to reposition the MIS-OLIF PEEK cage. When the reposition of the MIS-OLIF PEEK cage was performed, posterior instrumentation had to be removed as the first step for loosening of the intervertebral disc. Teeth on the surface of MIS-OLIF PEEK cage are designed for reducing the likelihood of expulsion. If the MIS-OLIF PEEK cage is inserted too deep, its teeth will lock with the edge of the vertebral body, preventing pull back of the MIS-OLIF PEEK cage. The surgical technique tips are important for complication, so to solve this problem, our technique should be considered. Reverse jack-knife position could open the contralateral disc space and the use of 2 retractor blade pins at upper and lower vertebral bodies should help to open the ipsilateral disc space. Longer retractor blade pins increase more distraction force. Bone removal may be required in case MIS-OLIF PEEK cage cannot be removed, but this may cause loosening of implant. Extraforaminal decompression of nerve root [23] as discussed above is the surgical option if we cannot directly reposition the MIS-OLIF PEEK cage. The disadvantages of this technique are more incision and more muscle dissection which need to be done. Another option of treatment is right side retroperitoneal approach and directly address to the MIS-OLIF PEEK cage. However, due to the MIS-OLIF PEEK cage was pointed to posterior of the right psoas muscle, where the nerve plexus is, so there is risk of nerve injury.

## 4. Conclusion

This study reported the complication from the deep and posterior position of PEEK cage in MIS-OLIF. The position and trajectory of MIS-OLIF PEEK cage are important during the insertion steps. It can cause contralateral nerve root compression and neurological complication. The surgical technique to proper pull the PEEK back into the acceptable position and relieve the pressure to the neural structure should be considered and prepared, since the designed implant may cause difficulty of removal. We described and discussed possible techniques to correct this unusual complication.

## References

[1] Pimenta L., Turner A. W. L., Dooley Z. A., Parikh R. D., Peterson M. D. (2012). Biomechanics of lateral interbody spacers: going wider for going stiffer. *The Scientific World Journal*.

[2] Ozgur B. M., Aryan H. E., Pimenta L., Taylor W. R. (2006). Extreme lateral interbody fusion (XLIF): a novel surgical technique for anterior lumbar interbody fusion. *The Spine Journal*.

[3] Ahmadian A., Verma S., Mundis G. M., Oskouian R. J., Smith D. A., Uribe J. S. (2013). Minimally invasive lateral retroperitoneal transpsoas interbody fusion for L4-5 spondylolisthesis: clinical outcomes. *Journal of Neurosurgery: Spine*.

[4] Castro C., Oliveira L., Amaral R., Marchi L., Pimenta L. (2014). Is the lateral transpsoas approach feasible for the treatment of adult degenerative scoliosis?. *Clinical Orthopaedics and Related Research^®^*.

[5] Mayer M. H. (1997). A new microsurgical technique for minimally invasive anterior lumbar interbody fusion. *Spine*.

[6] Davis T. T., Hynes R. A., Fung D. A. (2014). Retroperitoneal oblique corridor to the L2-S1 intervertebral discs in the lateral position: an anatomic study. *Journal of Neurosurgery: Spine*.

[7] Molinares D. M., Davis T. T., Fung D. A. (2016). Retroperitoneal oblique corridor to the L2-S1 intervertebral discs: an MRI study. *Journal of Neurosurgery: Spine*.

[8] Abe K., Orita S., Mannoji C. (2017). Perioperative complications in 155 patients who underwent oblique lateral interbody fusion surgery: perspectives and indications from a retrospective, multicenter survey. *Spine*.

[9] Jin J., Ryu K. S., Hur J. W., Seong J. H., Kim J. S., Cho H. J. (2018). Comparative study of the difference of perioperative complication and radiologic results: MIS-DLIF (minimally invasive direct lateral lumbar interbody fusion) versus MIS-OLIF (minimally invasive oblique lateral lumbar interbody fusion). *Clinical Spine Surgery*.

[10] Ohtori S., Orita S., Yamauchi K. (2015). Mini-open anterior retroperitoneal lumbar interbody fusion: oblique lateral interbody fusion for lumbar spinal degeneration disease. *Yonsei Medical Journal*.

[11] Silvestre C., Mac-Thiong J. M., Hilmi R., Roussouly P. (2012). Complications and morbidities of mini-open anterior retroperitoneal lumbar interbody fusion: oblique lumbar interbody fusion in 179 patients. *Asian Spine Journal*.

[12] Sato J., Ohtori S., Orita S. (2017). Radiographic evaluation of indirect decompression of mini-open anterior retroperitoneal lumbar interbody fusion: oblique lateral interbody fusion for degenerated lumbar spondylolisthesis. *European Spine Journal*.

[13] Phan K., Mobbs R. J. (2015). Oblique lumbar interbody fusion for revision of non-union following prior posterior surgery: a case report. *Orthopaedic Surgery*.

[14] Katzell J. (2014). Endoscopic foraminal decompression preceding oblique lateral lumbar interbody fusion to decrease the incidence of post operative dysaesthesia. *International Journal of Spine Surgery*.

[15] Kanno K., Ohtori S., Orita S. (2014). Miniopen oblique lateral L5-s1 interbody fusion: a report of 2 cases. *Case Reports in Orthopedics*.

[16] Gragnaniello C., Seex K. (2016). Anterior to psoas (ATP) fusion of the lumbar spine: evolution of a technique facilitated by changes in equipment. *Journal of Spine Surgery*.

[17] Chang J., Kim J. S., Jo H. (2017). Ventral dural injury after oblique lumbar interbody fusion. *World Neurosurgery*.

[18] Kubota G., Orita S., Umimura T., Takahashi K., Ohtori S. (2017). Insidious intraoperative ureteral injury as a complication in oblique lumbar interbody fusion surgery: a case report. *BMC Research Notes*.

[19] Lee H. J., Kim J. S., Ryu K. S., Park C. K. (2017). Ureter injury as a complication of oblique lumbar interbody fusion. *World Neurosurgery*.

[20] Youssef J. A., McAfee P. C., Patty C. A. (2010). Minimally invasive surgery: lateral approach interbody fusion. *Spine*.

[21] Fujibayashi S., Hynes R. A., Otsuki B., Kimura H., Takemoto M., Matsuda S. (2015). Effect of indirect neural decompression through oblique lateral interbody fusion for degenerative lumbar disease. *Spine*.

[22] Voyadzis J. M., Felbaum D., Rhee J. (2014). The rising psoas sign: an analysis of preoperative imaging characteristics of aborted minimally invasive lateral interbody fusions at L4-5. *Journal of Neurosurgery: Spine*.

[23] Papanastassiou I. D., Eleraky M., Vrionis F. D. (2011). Contralateral femoral nerve compression: an unrecognized complication after extreme lateral interbody fusion (XLIF). *Journal of Clinical Neuroscience*.

